# Encrusted Ureteral Stent in a Spanish Speaking Female: A Case of a Forgotten Stent Lost in Translation

**DOI:** 10.7759/cureus.5635

**Published:** 2019-09-12

**Authors:** Gagandeep S Gill, Tej J Desai, Shing-Yu Lin

**Affiliations:** 1 Medicine, Nova Southeastern University - Kiran C. Patel College of Osteopathic Medicine, Fort Lauderdale, USA; 2 Urology, Nova Southeastern University - Kiran C. Patel College of Osteopathic Medicine, Fort Lauderdale , USA; 3 Internal Medicine, AdventHealth East Orlando, Orlando, USA

**Keywords:** urology, forgotten ureteric stent, nephro-ureteral stent, medicine, disease prevention

## Abstract

Ureteral stents are used to establish patency in a non-draining ureter, as they are commonly placed in etiologies such as ureteral obstruction and urological surgery. One complication that occurs with stent placement is the absence of follow-up to remove the device. This may be due to a myriad of reasons, including non-compliance and lack of patient education. Forgotten stents can pose a dangerous scenario, as a retained stent can lead to urinary tract obstruction, urosepsis, and even kidney failure. In this study, we present a case of a Spanish-speaking patient with a retained ureteral stent who presented with left flank pain due to not understanding the need for stent follow-up.

## Introduction

A ureteral stent is a device that is commonly used to functionally re-establish the patency of a non-draining ureter. Indications for the placement of a ureteral stent include ureteral obstruction (from stones, tumors, or fibrosis), postoperative inflammation following ureteral repair or anastomosis, and prophylaxis for patients who undergo shock wave lithotripsy and have a high risk of obstruction due to large stones [1-2]. Ureteral stents are usually placed by a urologist with fluoroscopic guidance to ensure accurate placement.

Before the placement of the stent, however, patients are counseled on the nature of the procedure, possible stent-related symptoms, and complications. Complications associated with ureteral stent placement include hematuria, urinary tract infection, stent migration, and stent retention [3]. Of these, one complication that is entirely preventable is the retained/forgotten stent, which can be due to a combination of patient and surgical non-compliance. Retained stents can pose sometimes life-threatening complications due to their tendency to become encrusted or calcified [4]. Stent encrustation occurs secondary to the precipitation of calcium oxalate or uric acid on to the surfaces of the stent. Over time, severe encrustation of the ureteral stent can lead to urinary tract obstruction, sepsis, and eventually kidney failure [4]. Although urolithiasis, type of stent material, pregnancy, and the presence of bacteria are all risk factors for stent encrustation, the most important is the stent indwelling time. Removal of such stents requires surgical management with a combination of ureteroscopy and laser lithotripsy.

It is not only the responsibility of the patient but also of the surgeon to ensure that patients have appropriate follow-up so as to minimize complications. In the UK, in fact, forgotten ureteric stents accounted for the largest number of successful postoperative negligence claims [5]. We hereby report a case of a forgotten ureter stent with subsequent encrustation in a Hispanic female who did not fully understand what having a ureteral stent placed entailed.

## Case presentation

**Figure 1:**
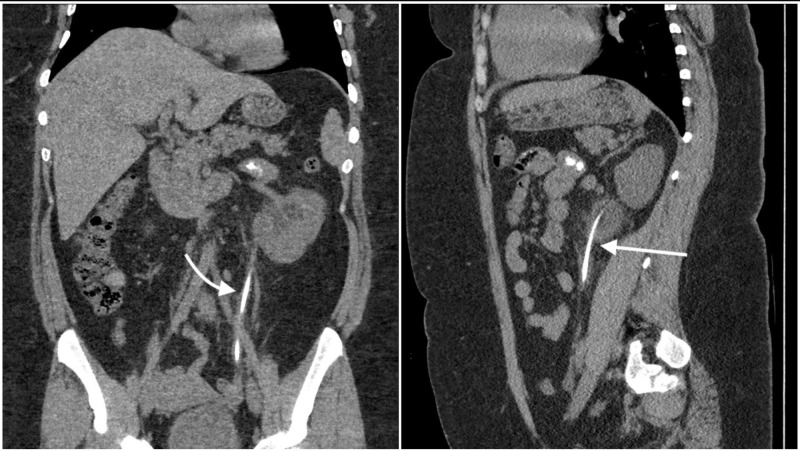
Coronal (left) and sagittal (right) view of the patient's abdominal CT showing an encrusted ureteral stent

A 29-year-old Spanish-speaking female, with no known medical history, presented to the emergency department with left flank pain and lower abdominal pain for the previous two weeks. Her associated symptoms included dysuria, nausea, and vomiting. She endorsed a history of kidney stones but denied fever, blood in emesis, diarrhea, or vaginal complaints. Laboratory findings at presentation were positive for an elevated white blood cell (WBC) count (11.21 103/uL) with elevated absolute neutrophils. Her urinalysis was found to be positive for 2+ blood, red blood cell (RBC) > 182/HPF, WBC>182/HPF, 1+ bacteria, and occasional triple phosphate crystals. Subsequent computed tomography (CT) imaging (Figure 1) of her abdomen revealed that she had an obstructed left renal collecting system with severe left hydronephrosis and a left-sided double-j ureteral stent. Upon extensive review of the patient’s records, it was found that the patient underwent a left ureteroscopy with stone extraction and stent placement over one-year prior at an outside facility. The patient reported that she was unaware that the stent required removal, and that she had done well until two weeks ago when her symptoms started. She was started on intravenous (IV) ceftriaxone, and urology was consulted for further surgical management.

A subsequent cystoscopy demonstrated a large stone involving the distal coil of her left ureteral stent. Extensive lithotripsy using both a cyber wand and a high-power holmium laser was used to fragment the stone and irrigate the stone fragments. Using fluoroscopic guidance, the stent was removed in an atraumatic fashion. Given the irregular ureteric findings on the intraoperative retrograde pyelogram, a 24-cm Polaris loop stent (Boston Scientific, Massachusetts, US) was advanced into the renal pelvis.

The patient was continued on antibiotics with a total of seven days (IV ceftriaxone x three days, Cefdinir oral x four days). Extensive discharge planning was discussed with the patient with an emphasis on the education of ureter stents and the importance of follow-up. The patient verbalized understanding and agreed to return for urology follow-up in one week for stent removal.

## Discussion

With the emergence of smaller, minimally invasive indwelling medical devices, the importance of follow-up is becoming increasingly critical. Anecdotally, as we saw in this case, patients who are fortunate enough to not experience symptoms until severe complications have occurred may be more inclined to forget about these medical devices and be lost to follow-up. Monga et al. (2011) have argued that patient education is the key to preventing forgotten ureteric stents [6]. Despite extensive counseling, up to 10% of their patients with retained ureter stents failed to have their stents removed and were lost to follow-up [6]. Therefore, patient education alone cannot be solely relied upon to ensure proper follow-up. Others have proposed that a system that actively engages patients is the best way to ensure patients’ understanding, this includes letters in the mail and automated messaging systems that periodically alert patients for follow-up [7].

Another strategy has been the development of stent registries that track the stent’s “status” from insertion all the way to removal. In these models, patients are provided with a tracking card that providers review periodically [7]. Unfortunately, studies have shown that stent registries such as this have proven ineffective when trying to prevent stent loss [7]. This has been primarily attributed to the fact that even when in place, clinicians reviewed the tracking cards much less frequently than required for them to be helpful. Although the tracking cards have proven to be largely ineffective, recent evidence has suggested that using an electronic tracking system can partially help to reduce the number of forgotten stents when compared to paper records [8]. In fact, Ather et al. found that by switching to a computer-based tracking system, they were able to reduce the rate of overdue stent removals by over 10% [9]. Building upon this concept, some institutions have gone as far as to propose to provide patients with wrist bands (with electronic bar codes) to serve as a visual reminder for patients, in addition to providing the ability to track stent status electronically [10].

Lastly, another significant issue contributing to forgotten stents and poor outcomes in today’s increasingly multicultural society is cultural and language barriers. A recent literature review by Caraway (2010) found that 66% of studies that examined poor health outcomes in Latino populations in the US determined that language barriers were the most significant attributing factor [11]. This case, where the patient was Spanish speaking and did not understand the instructions provided to her when her stent was originally placed is an example of this concept. With more than 13% of the US population speaking Spanish, especially in states like Florida, Texas, and California, it is imperative that providers educate patients in a language that they understand.

## Conclusions

Although this patient made a prompt recovery following the removal or her stent and subsequent antibiotic therapy, this is not always the case. Retained and encrusted ureteral stents can lead to life-threatening complications, including sepsis and death. Over the years, there have been various interventions to help reduce the number of forgotten stents but none have been completely successful in eradicating this avoidable complication. It is imperative that we develop newer, more effective strategies to ensure that ureteral stents get removed on time. Ultimately, in line with the duty to do no harm, it is both the patient’s and the physician’s responsibility to ensure that patients are provided with the appropriate education that they understand and receive adequate follow-up to avoid life-threatening ureteral stent retention.
